# Evolutionary dynamics in the fungal polarization network, a mechanistic perspective

**DOI:** 10.1007/s12551-017-0286-2

**Published:** 2017-08-15

**Authors:** Eveline T. Diepeveen, Leila Iñigo de la Cruz, Liedewij Laan

**Affiliations:** Department of Bionanoscience, Kavli Institute of NanoScience, Faculty of Applied Sciences, Delft University of Technology, P.O. Box 5046, 2600 GA Delft, the Netherlands

**Keywords:** Cell polarity, Protein network, Evolution, Fungi, Evolutionary conservation, Adaptation

## Abstract

Polarity establishment underlies proper cell cycle completion across virtually all organisms. Much progress has been made in generating an understanding of the structural and functional components of this process, especially in model species. Here we focus on the evolutionary dynamics of the fungal polarization protein network in order to determine general components and mechanistic principles, species- or lineage-specific adaptations and the evolvability of the network. The currently available genomic and proteomic screens in a variety of fungal species have shown three main characteristics: (1) certain proteins, processes and functions are conserved throughout the fungal clade; (2) orthologous functions can never be assumed, as various cases have been observed of homologous loci with dissimilar functions; (3) species have, typically, various species- or lineage-specific proteins incorporated in their polarization network. Further large-scale comparative and experimental studies, including those on non-model species representing the great fungal diversity, are needed to gain a better understanding of the evolutionary dynamics and generalities of the polarization network in fungi.

## Introduction

The asymmetrical accumulation of cellular components, such as organelles, proteins and cell-wall components, is fundamental for a proper completion of the cell cycle in both unicellular and multicellular organisms (Drubin 2000). The crucial step of symmetry breaking is observed during various cellular processes, such as cell division, cell motility, cell differentiation, cell–cell signaling and cell fusion (see, for example, Li and Bowerman 2010). The process of how cells break symmetry and polarize has been the focus of much attention in the literature and has been studied for over five decades. Polarity establishment has been traditionally studied in the budding yeast *Saccharomyces cerevisiae*. Budding yeast cells need to break symmetry, such as during each cell cycle in order to form a site of polarized cell growth and establish a bud that develops into a daughter cell. Much progress has been made in disentangling the structural and functional aspects of the components of this fundamental protein network. This wealth of information provides a valuable backbone for testing hypotheses on homology and for examining levels of conservation and divergence of the protein network members across different species. The remarkably diverse kingdom of fungi provides great opportunities to perform such comparisons, as it comprises an astonishing number of relative closely related species with a wide variety of cellular morphologies. The availability of various model organisms, such as the fission yeast *Schizosaccharomyces pombe*, the filamentous ascomycete *Ashbya gossypii* and the pathogenic basidiomycete *Ustilago maydis* or corn smut, facilitates direct comparisons of the functional and structural components of the machinery for cell polarity.

In this review we present: (1) an overview of the polarization machinery in the main fungal model species, (2) a discussion on the evolutionary dynamics of the network in fungi and (3) an overview of the current mechanistic understanding of the polarization machinery. Finally, we aim to link the discussed patterns of diversity, conservation and function to the great organismal diversity that we observe all around us, and recommend future directions of study.

## The polarization machinery of fungal model systems

**Fig. 1:**
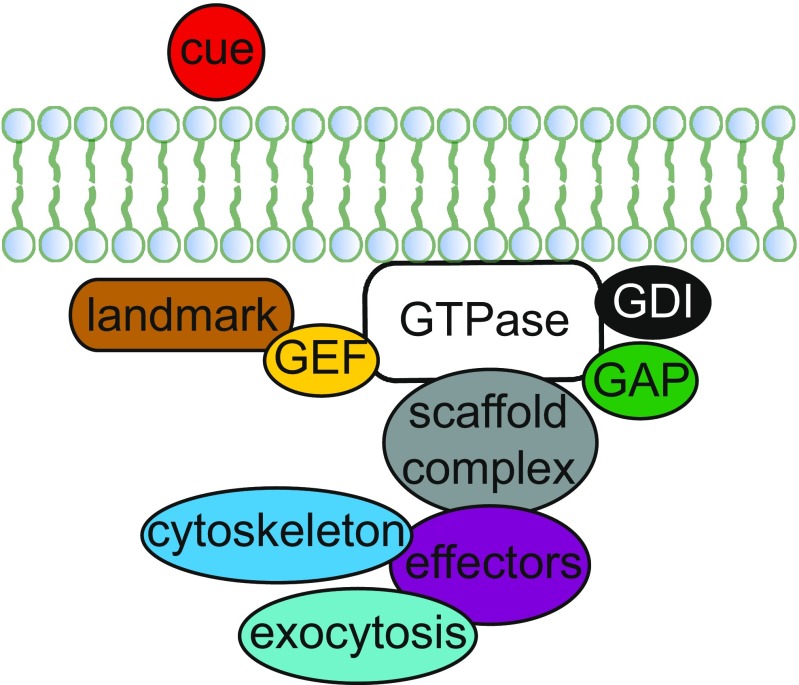
The key network of polarization. This cartoon gives an overview of the functional steps of cellular polarization. Briefly, polarity establishment is initiated by external or internal cues (shown here as an extrinsic* cue*;* in red*) resulting in landmark formation (*landmark*,* in brown*). The key regulator of polarity establishment and maintenance is a GTPase, which is regulated by its guanine nucleotide exchange factors (*GEF*,* in yellow*), GTPase-activating proteins (*GAP*,* in green*) and guanosine nucleotide dissociation inhibitors (*GDI*,* in black*). GTPase effectors (*effectors*, *in purple*) form a link between the scaffold protein complex (*scaffold complex*, *in gray*) and downstream processes, such as cytoskeleton organization (*cytoskeleton*, *in turquoise*) and exocytosis (*in blue*). For more information about the proteins involved in each step see Table 1. Note that the* size of the circles* do not represent protein concentrations in the cell or absolute protein (complex) sizes

The establishment of cell polarity is initiated by intrinsic or extrinsic cues, such as the marked site of previous cell division or mating pheromones in yeast, and results in the asymmetrical distribution of cell content. Polarity is essential to numerous processes during embryonic development, neural development and immunity throughout the Eukaryota (Chant 1999). In yeast this subcellular asymmetry is essential for proper cell division (e.g. budding) and mating (Chant 1999). Cellular content needs to be rearranged and allocated to the site of polarization during these processes. Actin patches and cables combined with myosin proteins facilitate this allocation and subsequent regional expansion. Proteins, vesicles, organelles and the cytoskeleton all relocate to the site of cytokinesis or mating. The nucleus (or nuclei, in the case of filamentous fungi) is oriented towards that site by microtubules. Two important steps are distinguished: (1) determining the direction of polarization, based on, for example, landmark proteins, pheromones, uneven distribution of mRNA; (2) the further establishment of the axis of polarization through activation of protein networks regulated by GTPases (Fig. 1). After these steps cell growth and subsequent cell division or mating take place. The signaling pathways involved in regulating these processes are hypothesized to be ancient and conserved, as they are found in all eukaryotes (Pruyne and Bretscher 2000). Molecular key players in these protein networks have been identified and characterized in detail in the budding yeast *Saccharomyces cerevisiae* (Table 1). Various excellent reviews, commentaries and seminal works are available on the details of budding yeast’s polarization protein networks (Pringle et al. 1995; Chant 1999; Pruyne and Bretscher 2000; Drees et al. 2001; Chang and Peter 2003; Pruyne et al. 2004; Park and Bi 2007; Bi and Park 2012; Martin and Arkowitz 2014). Here we give a brief overview of the main proteins, their functions and the main processes involved (Table 1).

**Table 1:**
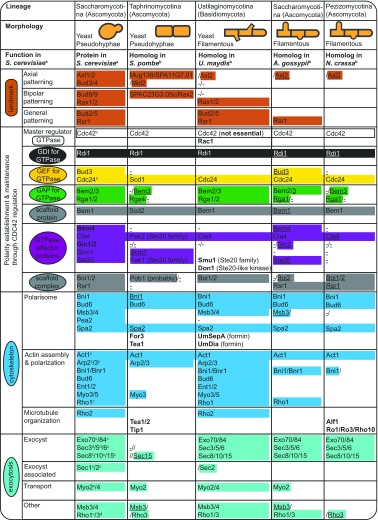
Overview of polarization proteins across fungal model systems

Proteins in **bold** represent species/lineage-specific polarization proteins and/or polarization proteins not found in *S. cerevisiae*. The minus symbol (−) represents proteins’ absence in examined studies. Empty lines represent cases for which no information was found in the examined literature. This synopsis is not exhaustive

^a^Following SGD; see main text for further references. Overall functional groupings (1st & 2nd column) as in Fig. 1. *S. cerevisiae* essential polarization proteins, Cdc28, Iqg1, Sec4 are non-evolvable (Liu et al. 2015)

^b^Following (Feierbach and Chang 2001; Pelham and Chang 2002; Chang and Peter 2003; Harris 2006; Banuett et al. 2008; Kaufmann 2008; Köhli et al. 2008; Bi and Park 2012; Martin and Arkowitz 2014); see main text for further references. Underlined (absence of) proteins represent findings of our recent survey (Diepeveen et al. 2017)

^c^Non-evolvable essential locus (Liu et al. 2015)

^d^Evolvable essential locus (Liu et al. 2015)

Landmark formation is a crucial step and depends on the state of the cell; haploid cells make use of the axial patterning proteins (i.e. Axl1, Axl2, Bud3, Bud4; Chant and Herskowitz 1991; Fujita et al. 1994; Halme et al. 1996; Roemer et al. 1996), while diploid cells make use of the bipolar patterning proteins (i.e. Bud8, Bud9, Rax1, Rax2; Zahner et al. 1996; Chen et al. 2000; Fujita et al. 2004). Both types of cells require a shared group of proteins consisting of Rsr1, Bud2, Bud5 (Bender and Pringle 1989; Chant et al. 1991; Chant and Herskowitz 1991; Park et al. 1993). This landmark establishment acts as a spatial memory of the precise location for the next cell cycle. Subsequently, there is a direct link between the landmark system and key regulators of polarization: the GTPase Cdc42 and its activator the guanine nucleotide exchange factor (GEF) Cdc24 (see Chant 1999 for hypotheses on proposed interactions). The signal transduction system for determining the polarization axis during mating is initiated by mating pheromones and consists of various proteins, such as Ste20, Ste5, Ste11, Ste7, Fus3, Ste12 and Far1 (Peter and Herskowitz 1994; Herskowitz 1995; Whiteway et al. 1995; Inouye et al. 1997; Leeuw et al. 1998; Pryciak and Huntress 1998). Observations of the authors of these studies reveal that different cues for different cell types exist and that these initiate different protein signal transduction pathways.

The polarization axis is still determined with equal efficiency even when mating or landmark cues are unavailable (e.g. Drubin 2000; Smith et al. 2013). Symmetry breaking can be kick-started by small fluctuations in concentrations away from homogeneity, which would otherwise be overruled by the aforementioned spatial cues. A switch-like mechanism controlled by the cyclin-dependent kinase Cdc28 then prevents untimely amplification of these ever-present fluctuations (Gulli et al. 2000; Li and Bowerman 2010; Klünder et al. 2013). For an overview of biophysical details see Box 1 in the Appendix.

To allocate cellular components to the site of budding or mating, the main axis for polarization then needs to be established and maintained, followed by polarization of the cytoskeleton. One of the most important steps is the localization and polarization of the master regulator of polarization, the GTPase Cdc42. Cdc42 interacts with many different proteins, including its own regulators (see, for example, Chant 1999; Martin 2015 for descriptions of the complex interconnectivity of Cdc42; Fig. 1; Table 1). There are three different groups of Cdc42 regulators: the GEFs (e.g. Cdc24 and the recently discovered Bud3) (Chant 1999; Pruyne and Bretscher 2000; Kang et al. 2014); the guanosine nucleotide dissociation inhibitors (GDI; e.g. Rdi1) (see Pruyne and Bretscher 2000; Martin 2015); GTPase-activating proteins (GAPs; e.g. Bem2, Bem3, Rga1, Rga2) (Kim et al. 1994; Zheng et al. 1994; Pringle et al. 1995; Stevenson et al. 1995; Chen et al. 1996; Chant 1999; Pruyne and Bretscher 2000; Drees et al. 2001; Smith et al. 2002; Chang and Peter 2003; Pruyne et al. 2004; Park and Bi 2007; Bi and Park 2012; Martin and Arkowitz 2014). These proteins control the (in)activation of active Cdc42 and dissociation from the cell membrane. Together with the important scaffolding protein Bem1, which mediates complex formation with Cdc42 and its GEF Cdc24, and, for example, Rsr1, these regulators help to spatially restrict the accumulation of active Cdc42 (Chant and Herskowitz 1991; Chenevert et al. 1992; Fujita et al. 1994; Zheng et al. 1995; Halme et al. 1996; Roemer et al. 1996; Park et al. 1997; Klünder et al. 2013). Cdc42 also interacts directly with a group of so-called effector proteins (Table 1). This group consists of Cla4, Ste20, Gic1, Gic2, Skm1 and Bem4. Bem4 interacts with various proteins, including Gic1, possibly localizing or regulating GTPases to the bud neck, and the major regulator of cell polarity Rho1 (see Zahner et al. 1996; Chen et al. 2000; Drees et al. 2001; Fujita et al. 2004). The polarization pathway has to stimulate the cytoskeleton and secretion systems to complete polarized cell growth. Boi1/2 are ligands of Bem1 and interact with various other proteins, such as Msb1 and Rho3, potentially forming a link between Cdc42 and proteins involved in, for example, exocytosis (Bender and Pringle 1989; Chant et al. 1991; Chant and Herskowitz 1991; Park et al. 1993; Matsui et al. 1996; Bender et al. 1996; Liao et al. 2013). The effector proteins Gic1 and Gic2 contribute to the polarization of the cytoskeleton (Chen et al. 1997; Brown et al. 1997; Chant 1999). Additional proteins connecting the polarization pathway to the cytoskeleton are, for example, Rho3, which directly targets Myo2 (Peter and Herskowitz 1994; Herskowitz 1995; Whiteway et al. 1995; Inouye et al. 1997; Leeuw et al. 1998; Pryciak and Huntress 1998; Robinson et al. 1999), and the Arp2/3 complex, which is involved in actin filament assembly (Machesky et al. 1994). The transportation of secretory vesicles to the site of polarization is controlled by a protein complex called the exocyst, consisting of, for example, Sec3 for membrane docking of vesicles and interaction with Rho1 (see, for example, Chant 1999). Interactions have been found for Rsr1, Bem1, Exo84 and Cdc24 with the exocyst subunit Sec15 (Drees et al. 2001). These complex protein–protein interactions ensure a proper establishment and maintenance of cell polarity.

Interestingly, the fundamental significance of budding yeast’s polarization machinery is supported by the identification of homologous proteins in non-fungal eukaryotes. The best example of homology, high-sequence similarity and functional conservation between distantly related species, is observed for Cdc42. This key regulator of polarization has been identified in hominids, rodents, teleost fishes and invertebrates, such as nematodes and fruit flies, among others (Johnson 1999; Cotteret et al. 2002). The signaling pathways underlying polarization are hypothesized to be common to all Metazoa. The PAR proteins, discovered in the nematode *Caenorhabditis elegans* (Kemphues et al. 1988), are conserved throughout the Metazoa (Goldstein and Macara 2007). The six *C. elegans* PAR proteins together with PKC3 and Cdc42 form a signaling network that is responsible for the asymmetric cell division observed in *C. elegans* zygotes (Goldstein and Macara 2007). Orthologous proteins for this network (with the exception of PAR2) have been identified in *Drosophila melanogaster*. A distant resemblance was only found for two PAR proteins in budding yeast (Goldstein and Macara 2007). A vertebrate GEF for Cdc42 has also been identified (Nishimura et al. 2006). These studies show that Cdc42 plays a major role in the establishment of polarization across a wide variety of organisms and therefore has an ancient origin.

**Fig. 2:**
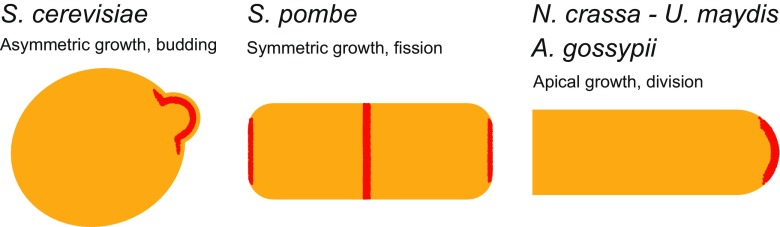
Overview of the different modes of polarization in different fungal cellular morphologies. Schematic representations of a budding yeast (*left*), fission yeast (*center*) and filamentous fungal (*right*) cell (or part thereof) are shown. The regions where polarization proteins accumulate during polarity establishment and cell division are highlighted in* red*. *Saccharomyces cerevisiae* forms a protrusion, referred to as a bud, at one side of the cell, which develops into a daughter cell. *Schizosaccharomyces pombe* grows at cell tips. Cells divide by medial fission to produce two daughter cells. The filamentous species grow at their tips, and cells are formed by septum formation (not shown). Note that *S. cerevisiae* and *S. pombe* are dimorphic and are able to form pseudohyphae in addition to the unicellular yeast state depicted here. For the dimorphic *Ustilago maydis* we only depict the filamentous state and ignore the budding yeast-like state this species has during the haploid phase

The polarization networks of various species of fungi have been examined (Table 1). Homologous proteins can be detected based on sequence similarity in respect to, for example, budding yeast proteins by means of genomic and/or proteomic screens, but orthologous functions of such proteins cannot be ensured without functional characterization. Various phenotypical distinctions, which potentially affect the polarization network and protein repertoire, can be made between species (e.g. unicellular vs. multicellular organism, symmetric growth, isotropic growth or asymmetric growth with a budding site; Fig. 2). The unicellular fission yeast *Schizosaccharomyces pombe* provides an excellent starting organism for comparison to determine the resemblances in cell polarity establishment between unicellular species. In both *S. pombe* and *S. cerevisiae* a specific site on the cell membrane is marked by landmark proteins in response to a cue, i.e. small GTPase proteins which are translocated to this spot and regulate actin cables and microtubules polarization towards the site of future growth. The new site of polarization is initiated based on the previous site of cell division in both species. Homologs of budding yeast’s landmark proteins Rax1/2 (SPAC23G3.05c/Rax2) have been identified in fission yeast, as have homologs for the axial budding proteins Bud4 (Mid2), Axl1 (Mug138) and Axl2 (SPA11G7.01) (see Martin and Arkowitz 2014; Table 1). Cdc42 is required for the establishment of cell polarization, and the Cdc24 ortholog Scd1 is the fission yeast’s GEF, with Gef1 as the second GEF (Coll et al. 2003). The Bem1 ortholog Scd2 might act as a scaffolding protein, although its exact role is not entirely clear (Chang et al. 1994). The Pob1 scaffolding protein has similarities to Boi1/2 and localizes For3 and the exocyst (Rincon et al. 2009; Nakano et al. 2011). Rdi1 is also present in fission yeast (see Martin and Arkowitz 2014). Although four different GAPs have been identified in budding yeast, only one GAP, Rga4, has previously been found in fission yeast, but in a recent survey we also identified a protein with homology to Bem3 (Diepeveen et al. 2017). Cdc42 effectors are the Ste20 family proteins Pak1/2 (Martin and Arkowitz 2014).

The fission yeast’s proteins Tea1, Tea2 and Tip1 have not been found in *Saccharomyces cerevisiae*’s symmetry breaking, but they do play critical roles in the spatial microtubule organization that marks the cell poles in *Schizosaccharomyces pombe* (Browning et al. 2000; Brunner and Nurse 2000). The Ras1 pathway plays an important role in regulating the polarization pathway. Ras1 activates Cdc42 (Martin and Arkowitz 2014). The fission yeast’s protein complex polarisome consists of Tea1, formin For3 and Bud6, with possible connections between For3 and Cdc42 and Rho3 (Chang and Peter 2003). Putative effectors of Tea1 are Pom1 and Tea3 (Nakano et al. 2002). Budding yeast has two formins, Bni1 and Bnr1, involved in the assembly of actin cables and rings (Evangelista et al. 1997). One specific aspect of cytokinesis that is substantially different between budding and fission yeasts is that in fission yeast the site of cell division is in the center of the cell and not at the periphery of the cell. Mid1 is a candidate as landmark protein for the positioning of the nucleus and contractile ring assembly (Chang and Peter 2003). These studies show that there are some striking similarities in key components and the core mechanism of the polarization network between the two yeast models, but at the same time the species are characterized by different modes of growth and cell division, which affects polarity establishment.

Non-yeast fungal species, such as *Ustilago maydis*, *Ashbya gossypii* and *Neurospora crassa*, have also been examined as key players and mechanisms of cell polarization (see Table 1). A remarkable difference between unicellular yeast (-like) species and filamentous fungi is that the latter are characterized by continuous polarized growth at the tips of their hyphae, thereby displaying specialized physiological aspects, such as tip-high calcium gradients (see, for example, Jackson and Heath 1993). The corn smut *U. maydis* is characterized by four possible states of polarized growth: yeast-like budding, filamentous structures in response to pheromones, tip-growth of newly formed dikaryotic hyphae outside the plant host and true filamentous structures inside the plant (see Banuett et al. 2008 and references therein). This fungus contains parts of the protein networks for, as an example, the recognition and interpretation of the landmark system (e.g. Axl2, Rax1/2, Rsr1, Bud2/5), polarity establishment (homologs of, for example, Cdc42, Cdc24, Bem3, Rga1/2, Cla4), exocytosis (e.g. Sec3/5/6/8/10/15) and actin (e.g. Arp2/3) and microtubule organization (e.g. Rho2) (Banuett et al. 2008). Interestingly budding yeast proteins such as Bud3/4/8/9 and Gic1/2 are missing, supporting earlier findings that these proteins are lineage-specific and restricted either to the Ascomycota or to the Saccharomycetaceae family (Diepeveen et al. 2017). Banuett also observed several polarization proteins not present in *Saccharomyces cerevisiae*; namely, the Rho GTPase Rac1, homolog of the mammalian Rac, has partially overlapping functions with Cdc42 in other filamentous fungi (see Banuett et al. 2008 for references) and is required for polarized growth. Even though homologs of proteins with functions in cell polarity in budding yeast are observed in *U. maydis*, they are not always characterized by orthologous functions. Most importantly, Cdc42 is not required for polarized growth in *U. maydis* (Mahlert et al. 2005).

The filamentous plant pathogen *Ashbya gossypii* also shows continuous growth at the tips of the filaments. New sites of polarization are initiated during hyphal growth, resulting in multiple polarity axes simultaneously (see Philippsen et al. 2005). Its genome has been screened for *Saccharomyces cerevisiae* homologs. One particular protein important for maintenance of hyphal growth, albeit not essential in *A. gossypii*, is Rho3 (Wendland and Philippsen 2001). The same authors also identified the presence of Cdc42, Cdc24, Rho1 (Wendland and Philippsen 2001), Bem2 (Wendland and Philippsen 2000) and Rsr1 (Bauer et al. 2004) in *A. gossypii*. Mutants for these loci show altered patterns or loss of polarity. Mutants of the polarisome proteins Bni1 and Spa2 also result in unusual patterns of growth and hyphal development, as discussed in Philippsen et al. (2005). Again, even though a group of *S. cerevisiae* proteins is found in *A. gossypii*, clear differences have been observed in how cells make use of them during symmetry breaking (see Philippsen et al. 2005). A recent study has found that the polyQ-containing protein Whi3 is an important regulator of symmetry breaking in *A. gossypii* (Lee et al. 2015). Whi3 aggregates and forms RNA–protein assemblies with Bni1 and Spa2 RNA molecules. This results in the asymmetrical synthesis of these important polarization proteins, thereby promoting symmetry breaking events.

Mutant screens in the red bread mold *Neurospora crassa* have lead to the identification of important elements that contribute to polarized growth in this ascomycetous fungus. These proteins are involved in, for example, actin (e.g., Act1) or microtubule cytoskeleton (i.e. Alf1, Ro1, Ro3, Ro10), components of signaling pathways (e.g. Cdc42, Cdc24, Bem1, Lrg1) and the secretory pathway (e.g. Sar1, Sec53) (Seiler and Plamann 2003). Interestingly, mutants show distinct differences in phenotype (e.g. overall or local effects) which have not been detected in unicellular yeast. Seiler and Plamann (2003 hypothesized that filamentous growth is more complex and has a more dynamic regulatory regime. These same authors also postulated that in light of *N. crassa*’s fast and constant hyphal growth at the tip, the secretory system is especially important in this species and the microtubular network is more complex. Homologs of budding yeast’s polarisome have been found, namely, Spa2, Bni1, Bud6 and also the exocyst components Sec3, 5, 6, 8, 10, 15 and Exo70,84 (see Riquelme et al. 2011). Various studies, especially those in *N. crassa*, stress the complex differences between model organisms with different phenotypes.

It has often been hypothesized that at the very least the overall mechanistic principles and cellular processes observed in *Saccharomyces cerevisiae* are conserved throughout the fungal clade and across the Eukaryota (Pruyne and Bretscher 2000; Wendland 2001; Nelson 2003). The overview in Table 1 illustrates this by means of various proteins present in all of the examined fungal model organisms. Work covering a broad spectrum of fungal species has focused on detecting orthologous of *S. cerevisiae* polarization proteins. Nevertheless, our brief overview of the mechanisms in various model species indicates that there are various vast differences in how organisms make use of the *S. cerevisiae* orthologs. All model species also have lineage- or species specific proteins that are not found in the other species (Table 1; Diepeveen et al. 2017), indicating a level of unique specialization. Recent progress in, for example, integrating molecular data into theoretical modeling (Box 1 in the Appendix) suggest a possibility of multiple polarization networks, which are hypothesized to be implemented differently in different species (Goryachev and Leda 2017). Although detailed and large-scale genetic and mutant screens are available in few fungal model species (*Neurospora crassa*: Seiler and Plamann 2003; *Aspergillus nidulans*: Li et al. 2006), further examination across non-model fungal species is needed to determine a more precise degree of homology. Other approaches that ultimately provide valuable insights are currently being developed, such as large-scale orthology prediction tools (T. Gehrmann, personal communication), the creation of genome-wide knock-out libraries and experimental studies, including experimental evolution and synthetic biology studies (minimal system, cell-rewiring to discover alternate or “bypass” mechanism). We currently have many individual and/or small-scale comparative findings, but an overall and detailed digest on the functionality, variability and conservation of the symmetry breaking mechanism across many species is still lacking.

## Evolutionary dynamics of the polarization protein network

How complex patterns are formed and how individuals adapt to their environments are key questions in cell and evolutionary biology. It has recently become possible to combine these questions and ask if, and if so, how the biomolecular networks that form these complex patterns affect the adaptation of an organism to its environment. With the availability of a variety of resources, such as genome databases, strain collections and gene-deletion databases, the budding yeast *Saccharomyces cerevisiae* and its close relatives are great model systems to study this topic. Questions related to how strains or species adapt to specific environments, especially members of the Saccharomycetaceae family, range from experimental evolution studies on laboratory strains (Segrè et al. 2006; Leu and Murray 2006; Gresham et al. 2008) to specifying characteristics, such as performance of wild species in fermentation (Boynton and Greig 2016). In relation to the fungal polarization network, our previous work focused on the evolutionary dynamics of the polarization network both within budding yeast and across the fungal phylogeny (Laan et al. 2015; Diepeveen et al. 2017; discussed in more detail in the following text). By combining the findings of the different approaches our aim here is to reveal patterns of evolutionary dynamics in this biomolecular network.

Various factors and processes that influence protein (network) evolution have been described (see, for example, Pál et al. 2006; Evlampiev and Isambert 2008; Zhang and Yang 2015 for a discussion of these factors). One group of causal factors is generated by the functional importance of proteins. Interestingly, the full *Saccharomyces cerevisiae* genome has been examined and tested for essential genes (Liu et al. 2015), with the results providing insights into whether a cell is viable or not without the locus under study. Essentiality is, however, strongly dependent on the genetic background and can be different not only in different strains or species, but also in different environmental conditions, such as growth medium. Liu et al. screened essential proteins for cellular evolvability (Liu et al. 2015). They found that approximately 9% of the approximately 1000 proteins identified as essential could in fact overcome the initial loss by adaptive evolution within 10 days. Various proteins connected to the polarization network are found to be essential in *S. cerevisiae* (Table 1) and are involved in different steps during symmetry breaking (see Fig. 1), such as timing (Cdc28), the key-regulator of polarization Cdc42, and its GEF Cdc24, actin assembly (Act1, Arp2/3) and exocytosis (Sec3/5/6/8/10/15, Exo70/84, Rho1/3). Most of these essential proteins are non-evolvable, and only Rho3 and Sec3/5 mutants can recover under standard conditions. These results indicate that evolvability is very different between members of the same cellular protein network.

In previous work we investigated the evolvability of the polarization network by examining how the polarization protein adapted to a strong genetic perturbation—in this case by means of inactivation of the nearly essential scaffolding protein Bem1. By deleting Bem1 we focused on adaptation to this specific cellular module and created a model system to obtain a mechanistic understanding of adaptation. First, we found that polarization is heavily disturbed in this model, with, as a result, decreases in growth rates by roughly eightfold. Bem1 binds directly to Cdc42 and forms the Bem1/Cdc42 complex by recruiting further proteins (see section The polarization machinery of fungal model systems; Lin et al. 2009). With Bem1 inactivated, the activation of Cdc42 by the GEF Cdc24 is hypothesized to be reduced. Interestingly, we found that yeast cells overcame this detrimental effect by inactivation of the GAPs Bem3 and Bem2, thereby reducing the inactivation of the available Cdc42. Another mechanism hypothesized to act at the same time is the inactivation of Nrp1, which was also observed. Nrp1 has a presumed function in the timing and initiation of polarization (Laan et al. 2015), which is brought forward by the actions of Cdc28 (Lew and Reed 1993). This protein stimulates the release of Cdc24 from the nucleus, which in turn activates Cdc42 (Shimada et al. 2000). These results indicate that the polarization protein network can adapt to perturbations within a few hundred generations and that it adapts by altering the regulation of the key regulator of polarization—Cdc42. As such, it would appear that the polarization network of *Saccharomyces cerevisiae* can overcome perturbations and relatively rapidly adapt to changes to key components by regulatory shifts.

We found that the polarization network is able to adapt under laboratory conditions. To study the relevance for evolution in the wild we asked how conserved the budding yeast’s polarization repertoire is across the fungal tree of life. In an attempt to answer this question we screened 200 fungal strains and species from four different phyla for 34 selected polarization proteins. We observed dynamic patterns in the sheer number of proteins per strain/species, in the sequence similarity of the proteins and in the overall prevalence of proteins (Diepeveen et al. 2017). The examined strains and species grouped into three main clusters (i.e. filamentous fungi, basal unicellular fungi and yeast-like fungi) based on protein repertoire size, lineage, lifestyle and genetic distance. There was a reduction in repertoire size in the filamentous basidiomycetes and a further reduction in the basal and unicellular lineages of the Cryptomycota and Microsporidia. Yeast-like fungi tended to have the highest number of proteins. Some remarkable patterns of high sequence similarity throughout the tree were seen for Cdc42, Rdi1, Sec4, and Rho3. A possible explanation for this observation is that all of these proteins, with the exception of Rdi1, are essential proteins—albeit in *Saccharomyces cerevisiae*— and that all four proteins are short proteins (i.e. <250 amino acids) and consist of a single domain. It has been hypothesized that basically all residue sites are functionally important and that selection is strong to prevent deleterious mutations. Another interesting factor is that we found a group of 16 proteins with a high prevalence in all ascomycetes and basidiomycetes. This group includes Cdc42 and its direct regulators and effectors, and also proteins such as Spa2, Rho3 and Sec4/15. Interestingly, over 95% of the species examined contained at least 75% of this core. We therefore hypothesize that polarity establishment by regulation of Cdc42 is conserved over the tree of life, but the set of proteins that performs this function varies between species.

## Concluding remarks: polarization protein network and organismal diversity

In this synopsis we focus on the evolutionary dynamics and the mechanisms underlying the polarization network across fungi. Much work has been performed in order to work towards a complete understanding of this highly complex protein network. But how far have we come? And what needs to be done to be able to mechanistically understand the observed conservation and variability of the network across species? Ultimately, we want to be able to link the profound amount of knowledge currently available to the biology of individual species. This means that we want to be able to explain a species’ morphology, physiology and ecology using the results of bio-informatics, proteomics, functional, experimental evolution and theoretical modeling studies.

To date, most of the work has been done in the budding yeast *Saccharomyces cerevisiae* in a wide array of fields. This species has become the ultimate model system to study the molecular key players and underlying mechanism of cell polarization. Influential studies that started describing normal budding behavior, polarity of cellular content and the effect of mutations on polarization and abnormal budding patterns started roughly five decades ago (Marchant and Smith 1968; Sloat and Pringle 1978; Borisy 1978). Subsequent decades were witness to progression in determining the members of the protein network (discussed in the section The polarization machinery of fungal model systems), the functional importance of individual proteins through, for example, knock-out studies (Arkowitz and Lowe 1997; Bi and Park 2012) and, more recently, the essentiality and/or evolvability of protein network members (Laan et al. 2015; Liu et al. 2015) and theoretical modeling of the regulatory mechanisms of the pathway (see Box 1 in the Appendix). Despite these overwhelming number of studies, even within *S. cerevisiae* questions remain unanswered. For example, not much is known on how the *S. cerevisiae* protein network adapts to changes to core-members of the network. We do know the phenotypic effects of knock-outs of all *S. cerevisiae* genes through the generation of tools such as the Yeast Deletion Project (Giaever et al. 2002). However, an increasing number of studies indicate that the essentiality of a gene is context dependent; a gene can be essential under one growth condition but not in another. Also, importantly, due to epistasis the essentiality of a gene can be dependent on the genetic background. Although tools such as the Biological General Repository for Interaction Datasets (thebiogrid.org) contain a vast amount of data on interactions between loci, and some insights on epistasis are available (Jasnos and Korona 2007), the complex interactions of loci under experimental or natural settings are still hard to predict. In order to further understand the evolvability of the network, experimental studies are needed to provide new insights. The study by Laan et al. (2015) also identified a locus with so far unidentified functions in the polarization network: Nrp1. This locus was found to be inactivated, and the authors hypothesized a role in the process of initiating polarization (Laan et al. 2015). This finding indicates that even in *S. cerevisiae* not all (indirect) players of Cdc42 regulation have been discovered. Ultimately, this means that even after five decades of work the polarization protein network and especially its epigenetic interactions are still not entirely uncovered in *S. cerevisiae*.

Other complexities arise with comparative genomics studies. Care should be taken when homologs are identified based on sequence similarity. Even when a species has a true ortholog to a *Saccharomyces cerevisiae* locus with demonstrated functions in the polarization in budding yeast, orthologous functions cannot be assumed. With the increased availability of genomic and proteomic datasets [e.g. deletion libraries (Giaever et al. 2002); the Saccharomyces Genome Database (SGD; Cherry et al. 2011)] it is becoming more feasible to perform large-scale comparative screens. Therefore, the development of sophisticated tools to reliably identify orthologs is essential. Furthermore, any findings of orthologs need to be functionally confirmed, as proteins with shared ancestry can have different functions or can be used differently in different species; for example, Cdc42 is not the master regulator of polarity in *Ustilago maydis* (Mahlert et al. 2005). Different fungal species are also characterized by entirely different groups of essential proteins (Seiler and Plamann 2003). Further, functional studies are indispensable because fully sequenced fungal genomes are frequently characterized by a relatively high level of loci without homologs in available fungal databases (Galagan et al. 2003). Such loci are not taken into account in most comparative studies. Functional screening of individual (non-model) species would therefore provide valuable information about such species- or lineage-specific proteins and adaptation.

The observation that different species make use of proteins in different ways may be seen as a sign of adaptive (protein) evolution. Each species has to constantly adapt to its current—and often changing—environment. Included in the fungal kingdom are species adapted to a highly diverse array of environments, from forest soil decayers to plant- or vertebrate-specialized parasites. The wide array of different specialists resembles the wide array of niches filled by fungi. With niche specialization comes diversifying morphologies. There are an estimated of up to 5.1 million fungal species (O'Brien et al. 2005), including different types of unicellular and multicellular organisms (Fig. 2), and species that switch back and forth between two or more lifestyles. Morphologies are highly distinct within each different lifestyle or lifephase; for example, the fruiting bodies of basidiomycetes (i.e., mushrooms) vary greatly in shape, size and color, and there is a great variety in shape and mode of division in unicellular fungi ( budding yeast, fission yeast, Microsporidia). So how can we link or explain the level of phenotypic diversity with patterns of variation and conservation, sequence similarity, identified orthologs and functional discrepancies in the polarization network? What part does the mode of polarization play in the adaptation of a species to its niche? If we want to be able to understand why this protein network comprises different members in different species and acts differently in different organisms, then we also need to understand why their morphologies are different.

Various aspects of the fungal diverse life strategies make it hard to link the polarization repertoire with specific phenotypes. Many different species are bi- or trimorphic, meaning that they can switch back and forth between unicellular to multicellular states depending on various environmental stimuli and/or stressors. The cell shapes of these different states are highly dissimilar and seem to be related to the specific environment the cell is in. For example, in nutrient-rich media, *Saccharomyces cerevisiae* grows by budding, while in nutrient-poor conditions they grow pseudohyphae. Furthermore, one big difference between the different life phases or morphologies is that cell growth by budding and pseudohyphae is cell cycle dependent, while hyphal growth is not. This means that there is continuous polarized growth in the hyphae, without cell separation, while polarized growth in yeast and pseudohyphae only takes place in the G1 phase of the cell cycle, followed by cell separation. This decoupling of polarized growth and cell cycle suggests a relaxed need of cyclin-dependent kinases, as Cdc28 is essential in *S. cerevisiae* (Liu et al. 2015). In addition, filamentous fungi do not need the *S. cerevisiae* chitin ring, which forms the site of cell separation, nor the associated proteins. A further distinction can be made in growth speed of filamentous fungi and the mechanism they use to polarize the cytoskeleton. Fast-growing fungi, such as *Ustilago maydis* and *Neurospora crassa* depend on the actin cytoskeleton plus microtubule dynamics, while the slower growing filamentous fungi *Ashbya gossypii* depends more on the actin cytoskeleton, as discussed by Arkowitz and Bassilana (2011). These different methods imply differences in sets of proteins regulating the specific mode of cytoskeleton organization.

Table 1 reveals some signs that there are a couple of yeast-specific polarization proteins, such as Gic1/2 and Bem4, and also of some putative filamentous-specific polarization proteins not found in unicellular fungi, such as Ro1/3/10 in *Neurospora crassa*. At the same time, not all unicellular or filamentous fungi possess these proteins, indicating that they are not crucial to that particular morphology. In fact, virtually all species seem to make use of a general pathway in which (1) a pre-existing or external signal leads to polarity establishment by GTPase(s) controlled by several regulators, effectors and scaffolding proteins, (2) the cytoskeleton is polarized, induced by the members of a polarisome and (3) exocytosis and endocytosis are needed at the site of growth (Fig. 1). The exact proteins that perform roles within these processes and feedback loops can differ and can be seen as a species’ adaptation. However, a number of proteins are present across species, and these proteins seem to be the (flexible) core of polarization (Diepeveen et al. 2017). This core does not appears to be specific to life stage or morphology as the proteins forming this core are found across species with different morphologies.

In conclusion, the evolutionary dynamics of the fungal polarization protein network include both a shared mechanism, represented by a core of shared proteins, and variation in the presence and/or importance of processes and network members that can sometimes be linked to function/phenotype. This conserved core of proteins offers opportunities to study the evolutionary dynamics of the polarization network in more detail, such as by testing orthology functionally. However, since the bulk of work has been performed in a handful of developed fungal model systems, which often are separated by high levels of divergence and have quite different lifestyles, validation of suggested patterns between the mechanism of polarization and morphology is needed. We urge for a deeper investigation of functional characterization in a wider variety of species and for further development of strong/reliable computational tools to support future large-scale comparative studies. The last five decades of research on the fungal polarization network have resulted in signals that the variability of the protein network might reflect the great diversity in lifestyles/morphologies observed in fungi. However, broader comparative approaches are needed to test the generality of these observations in order to be able to link the polarization networks to the species’ biology.

## Appendix 1: Box 1—theoretical molecular models of yeast cell polarity

Much progress has been made on the development of theoretical molecular models of yeast cell polarity. Here we give a brief introductory and non-exhaustive overview of the most commonly used models. For a broad and detailed discussion of theoretical modeling of shape and growth we refer the reader to Pelcé (2004). The aim of theoretical models is to describe spontaneous symmetry breaking during yeast polarization based on positive feedback loops for the activation and localization of Cdc42. These feedback loops are required to destabilize a spatially uniform state of the cell (Goryachev and Leda 2017). From the field of physics, we know that *spontaneous symmetry breaking is achieved when the symmetric state is no longer stable*. In the case of cell polarity, the behavior is very close to equilibrium phase transitions, where there is a critical value—for example, the cytoplasmic concentration of Cdc42 activator (Gulli et al. 2000) and/or Cdc42 inhibitor activity (Knaus et al. 2007), beyond which the spatially homogeneous state is unstable (Lo et al. 2014). Cdc42–GTP clusters have become the de facto criterion for judging whether a given set of experimental conditions allow or prevent cellular polarization. Historically, Cdc42 polarization in budding yeast has been described by two categorized pathways, namely, the actin pathway and the reaction–diffusion pathway. The actin pathway describes the polarization of Cdc42 mediated by protein transport on actin cables. However, as the exact cargo and function of the pathway is still under debate (Layton et al. 2011; Freisinger et al. 2013; Watson et al. 2014), constructing a realistic model for symmetry breaking via this pathway has been complicated. In the reaction–diffusion pathway, the spontaneous symmetry breaking conditions are fulfilled (Goryachev and Leda 2017) and can be linked to Cdc42 polarization in yeast cells (Irazoqui et al. 2003). Landmarks and sensory inputs can be added as spatially dependent initial conditions (for a review, see Chang and Peter 2003).

The necessary condition of a model that attempts to describe the spontaneous Cdc42 polarization in yeast is the ability to maintain a kind of “memory” from the initial condition by sustaining polarization long after the initial perturbation. To accomplish that condition, it is necessary that the rate of activation grows faster with increasing Cdc42–GTP than does the rate of inactivation (Goryachev and Leda 2017), assuming that the total cellular amount of Cdc42 is conserved during the transition. Theoretically, the only way to ensure this condition is by having multiple cooperating feedback loops, for example by having the local rates of activation/inactivation depend on the activity of Cdc42 itself (Lo et al. 2014). The mathematics behind the modeling approach always involves solving a coupled system of reaction diffusion equations, and a stability analysis of the steady state solution:

$$ {\displaystyle \begin{array}{l}\frac{\partial {P}_1}{\partial t}\left(x,t\right)={f}_1\left({P}_1\left(x,t\right),{P}_2\left(x,t\right),\dots, {P}_n\left(x,t\right),x,t;{k}_1,{k}_2,\dots, {k}_m\right)+{D}_1{P}_{1 xx}\hfill \\ {}\vdots \hfill \\ {}\frac{\partial {P}_n}{\partial t}\left(x,t\right)={f}_n\left({P}_n\left(x,t\right),\dots, {P}_1\left(x,t\right),{P}_2\left(x,t\right),x,t;{k}_1,{k}_2,\dots, {k}_m\right)+{D}_n{P}_{nxx}\hfill \end{array}} $$

where, *P*(*x*, *t*) is the time-evolution of the concentrations of the different proteins, which is given by reaction kinetics, *D*_*P*_ is a diagonal matrix encoding the diffusion constants for the proteins *P*(*x*,  *t*), *x* is a spatial coordinate, *t* is time and *k*_1_ , *k*_2_ ,  .  .  , *k*_*n*_ are the reaction-rate constants. *f* is a function that describes the local reactions. The term *P*_*nxx*_ refers to the second spatial derivative of the *n* -th protein concentration. Symmetry breaking is modeled in a spatially extended system accounting for the membrane as well as the cytosol. Different spatial dimensions are used to model the reaction diffusions equations according to how the proteins diffuse; for example, along a curve (one-dimension) (Smith et al. 2013), membrane surface (two dimensions) (Thalmeier et al. 2016) or cytoplasm (three dimensions) (Klünder et al. 2013).

Experimental cell biology studies (Kozubowski et al. 2008), the evolution of the polarity network as well as theoretical studies suggest that several feedback loops are simultaneously at play (Goryachev and Leda 2017) and that only a few mutations are necessary to switch between them (Laan et al. 2015).
